# A Systematic Review on Immediate Implant Placement in Intact Versus Non-Intact Alveolar Sockets

**DOI:** 10.3390/jcm14072462

**Published:** 2025-04-03

**Authors:** Axelle Ickroth, Véronique Christiaens, Jeremy Pitman, Jan Cosyn

**Affiliations:** Department of Periodontology and Oral Implantology, Faculty of Medicine and Health Sciences, Oral Health Sciences, Ghent University, C. Heymanslaan 10, B-9000 Ghent, Belgium

**Keywords:** dental implant, single tooth, immediate, intact sockets, non-intact sockets

## Abstract

**Objectives**: The primary objective of this systematic review was to compare IIP in intact versus non-intact sockets in terms of buccal bone thickness. **Methods**: Two independent reviewers carried out an electronic literature search in PubMed, Web of Science, Embase, and Cochrane databases as well as a manual search to identify eligible clinical studies up to June 2024. Randomized controlled trials (RCTs), cohort studies, and case series on intact and/or non-intact sockets were included for analysis. The primary outcome was buccal bone thickness (BBT). Secondary outcomes were vertical midfacial soft tissue level change, pink esthetic score (PES), implant survival and complications. This systematic review was conducted in accordance with the PRISMA guidelines. **Results**: After screening 1001 unique titles and conducting manual searches, 20 articles reporting on 525 implants (intact: 265; non-intact: 260) in the anterior maxilla with a follow-up of up to 120 months were selected. The overall study quality was low, especially for non-intact sockets since only two RCTs could be found, and none demonstrated a low risk of bias. Meta-analyses were not feasible due to a lack of direct comparisons, and heterogeneity in terms of surgical approach, soft tissue handling, and restorative approach. BBT ranged between 1.10 and 3.18 mm (intact) and 1.18 and 3 mm (non-intact). Vertical midfacial soft tissue level change ranged between −0.13 and −0.58 mm (intact) and −0.03 and −0.59 mm (non-intact). Pink esthetic scores ranged between 10.48 and 12.80 (intact) and 9.25 and 12.43 (non-intact). Implant survival exceeded 90% in all studies and was 100% in the vast majority of the studies. **Conclusions**: This systematic review suggests a similar outcome of IIP in intact and non-intact sockets. However, the overall low study quality, a lack of direct comparisons, and heterogeneity rendered the comparison highly biased. Future studies should be conducted to establish an evidence-based treatment approach for IIP in non-intact sockets.

## 1. Introduction

Immediate implant placement (IIP) is a favored treatment concept since it makes tooth replacement with an implant-supported restoration feasible within the shortest possible time. Because post-extraction bone remodeling cannot be mitigated by IIP [1,2], stringent case selection has always been advocated, initially limiting IIP to patients with intact, preferably thick buccal bone walls [3]. Over the years, measures to preserve hard and soft tissues have become part of IIP. A flapless approach is the first measure to preserve buccal bone since raising a flap compromises the blood supply of the fragile buccal bone wall at the time of extraction [4]. Grafting of the buccal bone gap is another measure that has become standard of care in IIP given its anti-resorptive properties and favorable cost–benefit ratio [5]. Soft tissue augmentation limits midfacial recession [6] and should therefore be considered in patients with high esthetic demands, in patients with a high smile line, or when treating sites at risk for midfacial recession [7]. Immediate provisionalization has also been shown to reduce midfacial recession [8], although the body of evidence is weaker. Even though these surgical and restorative measures contribute to a favorable outcome, implant positioning has shown to be most decisive. Immediate implants need to be installed at the apical and palatal aspect to avoid advanced midfacial recession [9]. Surgical experience seems to be key in this respect with experts inducing three times less midfacial recession at immediate implants than non-experts. Since suboptimal implant positioning can never be completely avoided when applying free-hand surgery, guided surgery should be promoted in the esthetic zone.

Mainly based on a lack of clinical studies in 2019, the European Federation for Periodontology discouraged IIP in severely damaged sockets [10]. Since 2019, the scientific community has shown a clear interest in research on IIP in non-intact sockets. However, a systematic review has never been published on the outcome of IIP in intact versus non-intact sockets. The objective of the present systematic review was to compare IIP in intact and non-intact sockets in terms of buccal bone thickness, vertical midfacial soft tissue level change, and esthetic outcome. Based on these outcomes, the results of the present systematic review have the potential to affect the decision-making process for IIP. In addition, they may identify needs for further research in order to clarify unresolved questions with respect to the treatment of non-intact alveolar sockets with immediate implants.

## 2. Materials and Methods

The systematic review was accomplished by applying the Preferred Reporting Items of Systematic Reviews and Meta-Analyses (PRISMA) as described by Moher et al. [11]. The protocol was initially registered in May 2023 in the International Prospective Register of Systematic Reviews (PROSPERO) at the UK’s National Institute for Health Research (NIHR), University of York, Centre for Reviews and Dissemination (CRD42023421051).

### 2.1. Objectives

The primary aim was to compare IIP in non-intact alveolar sockets to IIP in intact alveolar sockets in terms of buccal bone thickness. The research question of focus was the following: ‘In adult patients undergoing single implant placement in the esthetic area, will IIP in non-intact sockets as compared to IIP in intact sockets result in different buccal bone thickness?’

The PICO elements related to this focused research question were the following:
PatientAdult patients undergoing single implant placement in the esthetic area (from second premolar to second premolar in the maxilla or mandible).InterventionIIP in non-intact sockets (all types except type I, as classified by Chu et al. [12]).ComparisonIIP in intact sockets (type I socket as classified by Chu et al. [12]).OutcomeBuccal bone thickness (primary outcome), vertical midfacial soft tissue level change, pink esthetic score (PES), implant survival, and complications (secondary outcomes).

### 2.2. Eligibility Criteria

Inclusion criteria comprised the following:

- Single-arm data from randomized controlled trials (RCTs), cohort studies, and case series published in English.
- At least 18-year-old patients.
- Systemically healthy patients.
- Solitary immediate implant in incisor, cuspid, or premolar position in the maxilla or mandible.
- Studies reporting on titanium implants.
- Socket grafting/Guided Bone Regeneration (GBR) performed.
- Data on at least one outcome variable of interest.
- At least 6 months of follow-up.
- Study population of at least 10 patients.

Studies were excluded based on study design (case control studies, cross-sectional studies, letters to the editors, reviews).

Additional exclusion criteria were as follows:

- Acute infection at the extraction site.
- Reporting on zirconia implants.
- Reporting on alveolar socket shield technique.
- Reporting on patients taking medications/therapy affecting bone metabolism (i.e., bisphosphonates, radiation therapy).
- Reporting on patients with pathologies affecting bone metabolism (i.e., osteoporosis, osteopenia, rheumatoid arthritis).
- Reporting on implants placed in sites affected by tumors.
- Lack of information on whether augmentation procedures were performed or not.
- Impossible to separate intact from non-intact alveoli.
- Involving the application of any additional therapy that could have affected healing outcomes (e.g., use of healing enhancers, such as Platelet-Rich Plasma (PRP), Platelet-Rich Fibrin (PRF), growth factors).
- Insufficient information on the timing of implant placement after tooth extraction.

### 2.3. Information Sources and Search Strategy

Two independent reviewers (AI and VC) conducted an electronic literature search as well as a hand search to identify eligible clinical studies in Pubmed, Web of Science, Embase and Cochrane databases as well as a manual search using the following search algorithm:
Patientdental implant OR dental implant [MeSH Terms] OR dental implantation OR dental implantation [MeSH Terms] OR oral implant OR tooth implant OR tooth implantation
AND
fresh extraction socket OR immediate implant OR immediate insertion OR immediate installation OR immediate placement OR immediately placed OR immediately inserted OR immediately installed OR tooth extraction [MeSH Terms]Interventionbuccal bone dehiscence OR buccal bone plate dehiscence OR buccal bone wall dehiscence OR buccal dehiscence OR damaged OR dehiscence defect OR incomplete OR labial bone dehiscence OR labial bone plate dehiscence OR labial bone wall dehiscence OR labial dehiscence OR non-intact OR osseous defectComparisonintact alveolar socket OR intact alveolus OR intact bone wall OR intact buccal bone OR intact labial bone OR intact socket

The final search string included a combination of the following search items as follows: Patient AND (Intervention OR Comparison). In all databases the same search string was applied; however, in Web of Science, Embase, and Cochrane databases, MeSH terms were replaced by keywords.

The two reviewers (AI and VC) independently evaluated all studies on their eligibility based on the above-described inclusion and exclusion criteria. First, this was performed at the title level, then at the abstract level. If doubt arose at the title or abstract level, studies were further scrutinized at the next level to avoid overlooking appropriate studies. Articles that still qualified at the abstract level were printed, and full texts were read. Any disagreement that emerged during the full-text screening were resolved through discussion with a third reviewer (JC). To evaluate inter-rater reliability in selecting relevant articles, kappa coefficients were computed at title, abstract, and full text levels. Reference lists from the studies identified through the electronic search were reviewed for additional references. An attempt was made to identify gray literature by searching through the database of the U.S. National Library of Medicine (www.clinicaltrials.gov). Furthermore, a manual search was conducted in the following journals up to June 2024: *Journal of Clinical Periodontology, Journal of Periodontology*, *Clinical Implant Dentistry and Related Research*, Clinical Oral Implants Research, *Clinical Oral Investigations*, *International Journal of Oral & Maxillofacial Implants*, and *International Journal of Oral & Maxillofacial Surgery*.

### 2.4. Data Extraction

Two reviewers (AI and JP) independently extracted relevant data from the final set of included articles and created tables summarizing study characteristics and outcomes of interest. Disagreement was resolved through discussion with a third reviewer (JC). Authors of included studies were contacted by email to obtain missing or incomplete data. A response rate of 50% was seen.

### 2.5. Risk of Bias Assessment

Quality assessment of the included RCTs and non-randomized studies were independently performed by 2 reviewers (AI and VC) using either the Revised Cochrane Risk-of-bias Tool for Randomized Trials (RoB 2, as developed by Sterne et al. [13]), or the Newcastle Ottawa Scale (NOS) for Observational studies, as developed by Wells et al. [14] For the RoB2, the following criteria were scored: (1) bias arising from the randomization process, (2) bias due to deviations from intended interventions, (3) bias due to missing outcome data, (4) bias in the measurement of the outcome, (5) bias in the selection of the reported result. All five domains were judged as low, unclear, or high risk of bias. To assess inter-rater reliability in the risk of bias assessment, a weighted kappa coefficient was calculated. For the NOS, the following criteria were scored: (1) selection of the study groups, (2) comparability of the study groups, (3) outcome assessment of included non-randomized studies. The included non-randomized studies were labelled as poor, fair, or good quality based on their number of stars in each domain. To assess inter-rater reliability in quality assessment, weighted kappa coefficients were calculated.

### 2.6. Data Analysis

The data were mainly analyzed from a qualitative point of view. The quantitative data analysis was limited to the description of mean values and standard deviations for each outcome of interest. Apart from one multi-center RCT [7], the data on intact and non-intact sockets in IIP had to be extracted from different studies with varying design, surgical approach, soft tissue handling, and restorative approach. Hence, a pairwise meta-analysis and a network meta-analysis were not feasible.

## 3. Results

### 3.1. Search

**Figure 1:**
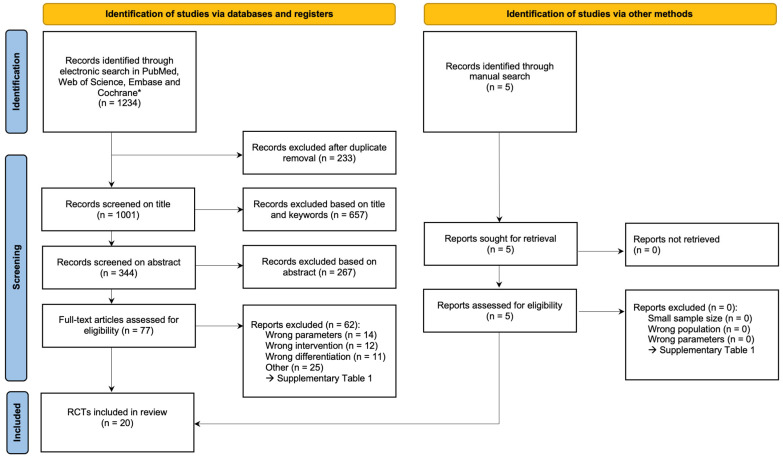
PRISMA flow chart on the search strategy.

The search strategy is illustrated in Figure 1. The electronic search returned 1234 titles in total (863 in PubMed, 304 in Web of Science, 56 in Embase, and 11 in Cochrane). After removing duplicates from the databases, a total of 1001 unique titles were screened. There was almost perfect agreement between both reviewers in the selection of appropriate studies at both title and abstract levels, given a kappa of 0.90 (*p* < 0.001) and 0.83 (*p* < 0.001), respectively. Sixty-two of the remaining seventy-seven articles were excluded after full text analysis. The manual search resulted in the inclusion of five additional articles. The reasons for exclusion are listed in Supplementary Table S1. Thus, 20 articles fully met the selection criteria for a qualitative and quantitative analysis [7,9,13,14,15,16,17,18,19,20,21,22,23,24,25,26,27,28,29,30].

### 3.2. Description of Selected Studies

Table 1 presents all relevant characteristics of the included studies. The analysis comprised data of 1 multi-center RCT comparing intact tot non-intact sockets [7], 9 studies on intact alveoli [9,15,18,19,20,21,22,26,27], and 10 studies on non-intact alveoli [16,17,23,24,25,28,29,30,31,32]. Seven studies were designed as RCTs [7,17,19,22,24,26,27], two as cohort studies [18,20] and the remaining eleven were case series [9,15,16,21,23,25,28,29,30,31,32]. All were prospective studies except for two on non-intact alveoli having a retrospective design [31,32]. In total, 525 patients received 525 single immediate implants (intact: 10 studies on 265 implants in 265 patients; non-intact: 11 studies on 260 implants in 260 patients) with a mean age ranging from 34.8 to 60 years. Follow-up ranged between 6 and 120 months. At the final study visit, 501 implants installed in 501 patients remained for evaluation (intact: 254 implants in 254 patients; non-intact: 247 implants in 247 patients) corresponding to an overall drop-out rate of 4.6%. All 20 studies installed implants in the anterior maxilla. The majority of the studies replaced incisors, canines, and premolars [7,9,16,17,18,19,20,21,30,31]. Three studies replaced incisors and canines [23,24,25], two replaced incisors and premolars [22,32], four studies only replaced incisors [15,27,28,29], and one study solely replaced premolars by means of a single implant [26]. Six studies included patients with a thin buccal bone wall [7,12,22,26,27,29], four reported solely on thick buccal bone walls [9,16,17,21], and in the remaining ten studies, the bony morphotype was not reported. In case of non-intact alveoli [7,16,17,23,24,25,28,29,30,31,32], all buccal bone defects were type II (a, b, or c) defects according to Chu et al. [12] with buccal bone dehiscence ranging from 2.9 mm to 9.9 mm. The lateral dimension of the buccal bone dehiscence was never reported. Surgery was performed with a flapless approach in nearly all studies except in six [21,23,25,26,29,32]. Six out of 20 studies performed soft tissue grafting with a connective tissue graft in all patients [9,22,24,27,29,31] or in some patients [9]. Three studies applied two-stage surgeries [24,25,27], one study applied one- and two-stage surgeries [29], while the remaining 16 applied one-stage surgeries. In 11 of these, immediate provisionalization was performed in all patients [7,9,15,17,18,20,21,22,27,31] or in some patients [29]. Concerning the type of bone graft used, ten studies reported on deproteinized bovine bone mineral (DBBM) [7,9,15,16,18,19,21,25,28,29], one study reported on collagen-enriched DBBM [22], four studies on allograft [20,26,27,30], four studies on a mixture of DBBM and autologous bone [17,23,24,31] and one study on bio-ceramics [32]. With respect to the use of a membrane, especially in non-intact sockets, only a few applied it, yet all were collagen membranes. One applied a membrane in intact sockets [26] and only six out of eleven applied a membrane in non-intact sockets [17,23,25,29,30,32].

### 3.3. Risk of Bias Assessment

Table 2 illustrates the risk of bias assessment of all included RCT studies using the RoB 2 tool [7,17,19,22,24,26,27]. None of the studies showed an overall low risk of bias. Five studies showed some concerns leading to an overall unclear risk of bias [7,17,19,22,27]. In three of the latter, some concerns were raised due to missing data or a lack of data reporting leading to a potential risk of reporting bias [7,17,22]. Two showed bias due to derivations from the intended interventions [19,27] and one showed additional bias arising from the randomization process [19]. Two RCTs showed an overall high risk of bias: one due to derivations from the intended interventions [24], the other due to using inappropriate methods to measure the outcome [26]. There was substantial agreement between both reviewers in the risk of bias assessment, given a weighted kappa of 0.660 (95% CI [0.381–0.934], *p* < 0.001).

Table 3 illustrates the risk of bias assessment of all non-randomized studies using the Newcastle Ottawa Scale (NOS) for observational studies. Three were designed as cohort studies [18,20,28] and the remaining ten were case series [9,15,16,21,23,25,29,30,31,32]. Two cohort studies demonstrated a good overall quality, given a score of seven stars [18] and eight stars [28]. The remaining eight studies scored poorly, with scores ranging from four stars [30] to six stars [9,15,16,20,21,23,25,29,31,32]. The low scores were primarily due to the absence of a control group. There was substantial agreement between both reviewers in the risk of bias assessment, given a weighted kappa of 0.555 (95% CI (0.365–0.745), *p* < 0.001).

### 3.4. Primary Outcome Variable: Buccal Bone Thickness

Four studies involving 124 implants in 124 patients reported on buccal bone thickness (BBT) in intact alveoli [9,20,22,26] and seven studies involving 167 implants in 167 patients reported on BBT in non-intact alveoli [16,23,24,25,29,30,32] (Table 4). In all studies, measurements were performed on midsagittal slices at the level of the implant shoulder. Considering single-arm data on intact alveoli, BBT ranged between 1.10 and 3.18 mm. For non-intact alveoli, BBT ranged between 1.18 and 3.00 mm.

### 3.5. Secondary Outcome Variables

#### 3.5.1. Vertical Midfacial Soft Tissue Level Change

Data of one multi-center RCT enabled a direct comparison of vertical midfacial soft tissue level change between intact and non-intact sockets [7] (Table 4). Intact and non-intact sockets showed a mean recession of −0.43 mm and −0.65 mm, respectively. The difference was not significant. Seven studies reported on vertical midfacial soft tissue level change in intact sockets [9,15,18,19,21,22,27]. One out of seven studies showed a vertical gain in midfacial soft tissue level by 0.20 mm at 12 months [27], whereas all others showed midfacial recession between −0.13 mm and −0.58 mm. Four studies reported on vertical midfacial soft tissue level change in non-intact sockets [23,24,25,29], all resulting in midfacial recession between −0.03 mm and −0.59 mm.

#### 3.5.2. Pink Esthetic Score

Eight studies reported on the PES for intact sockets [9,15,18,19,20,21,22,27] (Table 4). All of them used the original index generating a score on a total of 14 [34], which ranged from 10.48 to 12.80. Seven studies reported on the PES for non-intact sockets [17,23,24,28,29,31,32]. Meijer et al. [24] and Qian et al. [29] used a modified index generating a score on a total of 10 [33] pointing to 7.40 and 9.17, respectively. For the remaining five studies, PES ranged between 9.25 and 12.43.

#### 3.5.3. Implant Survival

Almost all studies reported on implant survival. Data of one multi-center RCT enabled a direct comparison of implant survival between intact and non-intact sockets [7] (Table 4). One out of 18 implants installed in intact sockets failed to integrate and was removed, leading to 94% survival, compared to 100% implant survival in 12 patients with a non-intact socket. Concerning single-arm data on intact alveoli, six studies reported 100% implant survival with a follow-up up to 24 months [15,18,19,22,26,27]. Longer studies reported 96% survival over 36 months [21] and 90% over 120 months [9]. For single-arm data on non-intact alveoli, nine out of ten included studies reported on implant survival. All studies reported 100% survival over a follow-up of up to 120 months [17,23,24,25,28,29,30,31,32].

#### 3.5.4. Complications

Three studies described complications in intact sockets (Table 4). Two reported on technical complications, one provisional crown fracture [12] and one permanent crown loosening [21]. Seyssens et al. [9] showed 33% peri-implant mucositis and 5.6% peri-implantitis over a period of 120 months. Three studies reported no complications [15,22,26]. Three studies described biological complications in non-intact sockets. Borgia et al. [17] showed 24.2% peri-implant mucositis over 12 months, Liu et al. [23] reported early wound healing complications (exudation and pyogenic infection) in five patients, and Meijer et al. [24] reported 20% peri-implant mucositis over 120 months. The latter additionally reported two technical complications, one permanent crown loosening and one permanent crown fracture. Only one study reported zero complications in non-intact sockets [28].

## 4. Discussion

The objective of the present systematic review was to compare IIP in intact and non-intact sockets in terms of buccal bone thickness, vertical midfacial soft tissue level change, and esthetic outcome. To the best of our knowledge, such a comparison has never been investigated yet could reveal relevant information affecting the decision-making process for IIP. In addition, the result of the present study may identify needs for further research in order to clarify unresolved questions with respect to the treatment of non-intact alveolar sockets with immediate implants.

A total of 20 studies, including RCTs, cohort studies, and case series, were identified through electronic and manual searches and met the selection criteria. Studies not applying grafting of the buccal bone gap were excluded, since this approach has become standard of care in IIP [10,35]. Indeed, socket grafting reduces buccal bone resorption and midfacial recession with a favorable cost–benefit ratio [5]. State-of-the-art IIP includes flapless surgery [4,36], guided implant placement [37], application of a bone grafting material [5,36] and CTG [6,36,38], and immediate provisionalization [8]. These concepts aim to preserve both hard and soft tissues while ensuring stable, long-term esthetic and clinical outcomes [35]. Only two of the included studies managed to implement all these concepts, hereby strictly adhering to the state of the art. However, these consisted of one RCT [22] with some concerns and one case series [31] of poor quality. The overall study quality was low, especially for non-intact sockets, since only two RCTs could be found, and none demonstrated a low risk of bias. Apart from one multi-center RCT [7], direct comparisons between intact and non-intact sockets were not available in the literature. The search highlighted significant variability in study design, surgical approach, soft tissue handling, and restorative approach, which precluded meta-analyses and necessitated a descriptive quantitative analysis. Therefore, the results of the present study should be considered as an exploration of IIP in non-intact sockets.

Buccal bone thickness was reported in 11 studies, with a range of 1.10–3.18 mm in intact sockets and 1.18–3.0 mm in non-intact sockets. While these values suggest comparable outcomes across the two groups, the lack of direct comparisons between intact and non-intact sockets prevents robust conclusions. Prospective cohort studies are required to compare BBT between intact and non-intact sockets at immediate implants. Since BBT may be reduced by a buccal implant position, information on implant positioning would have been valuable. Interestingly, only one study reported such information [6]. To overcome suboptimal implant placement, guided surgery has been advocated [10,35]. Unfortunately, only eight of the included studies mentioned the use of a surgical guide [7,17,18,19,22,24,25,31]. Four of them used pilot drills [7,18,19,24], and one study used computer-guided surgery [31].

Midfacial recession was slightly higher in non-intact sockets (mean: −0.65 mm) than in intact sockets (mean: −0.43 mm) in one direct comparison, although the difference was not significant [7]. However, in all studies, mean midfacial recession ranged between −0.13–−0.58 mm (intact) and −0.03–−0.59 mm (non-intact), which may be considered low and does not necessarily impact clinical decision-making. On the other hand, extreme values may also result in acceptable mean midfacial recession [9], which calls for the reporting of frequency distributions in clinical studies. A buccal bone dehiscence was identified as a risk factor for midfacial recession in the same study, irrespective of the timing of implant placement (immediate or delayed) [7]. Single-arm data from other studies (seven on intact sockets, four on non-intact sockets) reported acceptable midfacial recession, all pointing to a maximum of −0.6 mm in both groups. It is important to note that the majority of the included studies neither applied a CTG in conjunction with IIP [7,15,16,17,18,19,20,21,23,25,26,28,30,32] nor installed an immediate provisional crown [16,19,23,24,25,26,28,29,30,32]. Both have been shown to contribute to midfacial soft tissue stability [6,8]. In fact, the combination of CTG and immediate provisionalization was only applied in three studies pertaining to intact sockets [9,22,27] and one study pertaining to non-intact sockets [31]. Hence, the state of the art in IIP was seldom applied in the included studies. From this information, it becomes clear that an evidence-based treatment approach for IIP in non-intact sockets has not been established. Future studies should elucidate the potential impact of the vertical and lateral dimension of a buccal bone dehiscence on midfacial recession, the need for a membrane, and the need for soft tissue augmentation. Since soft tissue augmentation performed at the time of IIP may have an impact on the outcome of socket grafting when dealing with a non-intact socket, clinical studies on procedural aspects and timing are required.

From an esthetic point of view, similar outcomes in intact and non-intact sockets were found when considering the data of the original PES. Intact sockets demonstrated mean PES scores between 10.48 and 12.80, while mean PES scores ranged from 9.25 to 12.43 in non-intact sockets. Direct comparisons were not available, again making robust conclusions impossible. Still, the present findings seem to indicate that a favorable esthetic outcome is possible following IIP in non-intact sockets, which is reassuring.

High implant survival rates were reported for intact as well as for non-intact sockets, with most studies achieving 100% implant survival over follow-up periods of up to 120 months.

In contrast to implant survival, complications were seldom described in intact as well as in non-intact sockets. Due to such underreporting, any comparison in terms of the risk for peri-implant pathology would be speculative at this time.

When comparing the findings of this systematic review to earlier published systematic reviews, similar outcomes were found. Two recent systematic reviews of Amid et al. [39] and Chen et al. [40] studied implant survival and clinical outcomes of IIP in damaged sockets, yet without comparing them to those in intact sockets. The outcomes for implant survival (96.8% and 98.1%, respectively), midfacial recession (−0.49 mm and −0.25 mm, respectively), and PES (8.93 and 12.34, respectively) after 12 months were in line with the mean outcomes of the non-intact group in the current paper. Both systematic reviews concluded that IIP in non-intact sockets was feasible. Another recent systematic review by Campi et al. [41] compared IIP in non-intact sockets to healed sites, concluding that IIP in damaged sockets could achieve comparable clinical and radiographical outcomes compared to delayed implant placement.

When interpreting the results of this systematic review, the following limitations should be considered. First, the overall quality of the evidence was low, especially for non-intact sockets, with only two RCTs found. For intact sockets, four RCTs could be included. One RCT pertaining to intact sockets and one RCT pertaining to non-intact sockets yielded a high risk of bias, whereas none demonstrated a low risk of bias. Given the overall low study quality, definitive conclusions cannot be drawn. Second, meta-analyses were not feasible due to a lack of direct comparisons and heterogeneity in terms of surgical approach, soft tissue handling, and restorative approach. This resulted in a descriptive quantitative analysis. As a result, the results of the present systematic review should be considered as an exploration of IIP in non-intact sockets.

Future studies should be conducted to establish an evidence-based treatment approach for IIP in non-intact sockets, clarifying the potential impact of the vertical and lateral dimension of a buccal dehiscence on midfacial recession, the need for a membrane, and the need for soft tissue augmentation. Finally, the method and timing of soft tissue augmentation needs to be studied in non-intact sockets.

## 5. Conclusions

This systematic review suggests a similar outcome of IIP in intact and non-intact sockets. However, the overall low study quality, a lack of direct comparisons, and heterogeneity render a comparison highly biased. Prospective cohort studies are required to compare the outcome of IIP in intact and non-intact sockets. In addition, RCTs need to be conducted to establish an evidence-based treatment approach for IIP in non-intact sockets.

## Figures and Tables

**Table 1:** Study characteristics.

	**Author**	**Study Design, Setting**	**Follow-Up** **(Months)**	**No. Patients/Implants**	**Mean Age (y)**	**Implant Positions** **(Max/Mand I–C–P)**	**CTG (Yes/No)**	**One/Two-Stage Surgery**	**Immediate Provisionalization (Yes/No)**	**Drop-Outs**	**Bone Graft Type**
**Comparative clinical studies**	Cosyn et al., 2024 intact [7]	RCT, U+P	6	18/18	53.1	Max 10 I–1 C–7 P	No	One-stage	Yes	0	DBBM
	Cosyn et al., 2024 non-intact [7]	RCT, U+P	6	12/12	53.1	Max 6 I–6 P	No	One-stage	Yes	0	DBBM
**Single-arm data on intact alveoli**	Arora & Ivanovski, 2016 [15]	Case series, P	24	18/18	NR	Max 18 I	No	One-stage	Yes	0	DBBM
	Cardaropoli et al., 2015 [18]	Cohort, P	12	26/26	42.4	Max I–C–P	No	One-stage	Yes	0	DBBM
	Cardaropoli et al., 2022 [19]	RCT, P	12	24/24	57.8	Max I–C–P	No	One-stage	No	0	DBBM
	Chu et al., 2021 [20]	Cohort, U+P	12	48/48	58.1	Max 25 I–5 C–8 P	No	One-stage	Yes	0	ALLO
	Cosyn et al., 2011 [21]	Case series, U	36	30/30	54	Max 19 I–2 C–9 P	No	One-stage	Yes	5	DBBM
	Cosyn et al., 2024 [22]	RCT, U	12	40/40	55.9	Max 25 I–15 P	Yes	One-stage	Yes	2	c-DBBM
	Naji et al., 2021 [26]	RCT, P	6	14/14	40.2	Max 14 P	No	Two-stage	No	0	ALLO
	Puisys et al., 2022 [27]	RCT, P	12	25/25	45.8	Max 25 I	Yes	One-stage	Yes	0	ALLO
	Seyssens et al., 2020 [9]	Case series, P	120	22/22	50	Max 17 I–1 C–4 P	7/22 Yes	One-stage	Yes	4	DBBM
**Single-arm data on non-intact alveoli**	Assaf et al., 2017 [16]	Case series, U	12	14/14	57.1	Max 7 I–3 C–4 P	No	One-stage	No	0	DBBM
	Borgia et al., 2022 [17]	RCT, U+P	12	31/31	53.4	Max 23 I–3 C–5 P	No	One-stage	28/31 Yes	3	MIX
	Liu et al., 2019 [23]	Case series, U	12	45/45	36.7	Max 39 I–6 C	No	One-stage	No	4	MIX
	Meijer et al., 2024 [24]	RCT, U	120	20/20	43.7	Max 17 I–3 C	Yes	Two-stage	No	5	MIX
	Mizuno et al., 2022 [25]	Case series, U	12	20/20	60	Max 19 I–1 C	No	Two-stage	No	0	DBBM
	Pohl et al., 2022 [28]	Case series, P	12	10/10	55.1	Max I	No	One-stage	No	0	DBBM
	Qian et al., 2023 [29]	Case series, U	12	12/12	34.8	Max I	Yes	Both	2/12 Yes	0	DBBM
	Sarnachiaro et al., 2016 [30]	Case series, U	6-9	10/10	NR	Max 2 I–3 C–5 P	No	One-stage	No	0	ALLO
	Sicilia-Felechosa et al., 2020 [31]	Case series, P	Mean 3.44 years	40/40	58.7	Max 26 I–1 C–13 P	Yes	One-stage	Yes	1	MIX
	Zhao et al., 2023 [32]	Case series, U	60	46/46	37.8	Max 39 I–7 P	No	One-stage	No	0	BIO-CERA

U–P: University setting–Private practice; Max/Mand–I–C–P, Maxilla/Mandible–Incisor/Canine/Premolar; DBBM–c-DBBM–ALLO–MIX–BIOCERA, Deproteinized Bovine Bone Mineral–collagen-enriched DBBM–Allograft–Mixture of DBBM and autogenous bone–Hydroxyapatite Bio-ceramic; NR, Not reported.

**Table 2:** Revised Cochrane Risk-of-bias Tool for Randomized Trials (RoB 2). Color codes indicate a low risk of bias (green), some concerns (yellow), and a high risk of bias (red) in all five domains. The last column illustrates the overall risk of bias of included RCTs.

	D1	D2	D3	D4	D5	
Borgia 2022 [17]						
Cardaropoli 2022 [19]						
Cosyn 2024 [7]						
Cosyn 2024 [22]						
Meijer 2024 [24]						
Naji 2021 [26]						
Puisys 2022 [27]						

**Table 3:** Newcastle Ottawa Scale (NOS) for Observational studies.

Author	Selection				Comparability	Outcome			Total	Overall Quality
	Representativeness of the exposed cohort/case (Maximum: **✵**)	Selection of the non-exposed cohort/case (Maximum: **✵**)	Ascertainment of exposure (Maximum: **✵**)	Outcome of interest not present at study start (Maximum: **✵**)	Comparability of cohorts on basis of design or analysis (Maximum: **✵✵**)	Assessment of outcome (Maximum: **✵**)	Follow up long enough for outcomes to occur (Maximum: **✵**)	Adequacy of follow-up of cohorts (Maximum: **✵**)		
Arora & Ivanovski, 2016 [15]	**✵**		**✵**	**✵**		**✵**	**✵**	**✵**	6	Poor
Assaf et al., 2017 [16]	**✵**		**✵**	**✵**		**✵**	**✵**	**✵**	6	Poor
Cardaropoli et al., 2015 [18]	**✵**		**✵**	**✵**	**✵**	**✵**	**✵**	**✵**	7	Good
Chu et al., 2021 [20]	**✵**		**✵**	**✵**		**✵**	**✵**	**✵**	6	Poor
Cosyn et al., 2011 [21]	**✵**		**✵**	**✵**		**✵**	**✵**	**✵**	6	Poor
Liu et al., 2019 [23]	**✵**		**✵**	**✵**		**✵**	**✵**	**✵**	6	Poor
Mizuno et al., 2022 [25]	**✵**		**✵**	**✵**		**✵**	**✵**	**✵**	6	Poor
Pohl et al., 2022 [28]	**✵**	**✵**	**✵**	**✵**	**✵**	**✵**	**✵**	**✵**	8	Good
Qian et al., 2023 [29]	**✵**		**✵**	**✵**		**✵**	**✵**	**✵**	6	Poor
Sarnachiaro et al., 2016 [30]	**✵**		**✵**	**✵**				**✵**	4	Poor
Seyssens et al., 2020 [9]	**✵**		**✵**	**✵**		**✵**	**✵**	**✵**	6	Poor
Sicilia-Felechosa et al., 2020 [31]	**✵**		**✵**	**✵**		**✵**	**✵**	**✵**	6	Poor
Zhao et al., 2023 [32]	**✵**		**✵**	**✵**		**✵**	**✵**	**✵**	6	Poor

**✵** Allocation of stars as per rating sheet: A study can be given a maximum of one star for each numbered item within the Selection and Outcome categories. A maximum of two stars can be given for Comparability.

**Table 4:** Study outcomes.

	**Author**	**Buccal Bone Thickness (mm)** **Mean (SD)**	**Vertical Midfacial Soft Tissue Level Change (mm)** **Mean (SD)**	**Pink Esthetic Score** /10 */14 # (SD)	**Implant Survival** **%**	**Complications**
**Comparative clinical studies**	Cosyn et al., 2024 intact [7]	NR	−0.43 (0.44)	NR	94%	NR
	Cosyn et al., 2024 non-intact [7]	NR	−0.65 (0.30)	NR	100%	NR
**Single-arm data on intact alveoli**	Arora & Ivanovski, 2016 [15]	NR	−0.22 (0.83)	10.78 # (1.93)	100%	NR
	Cardaropoli et al., 2015 [18]	NR	−0.21 (0.32)	11.46 # (1.45)	100%	0
	Cardaropoli et al., 2022 [19]	NR	−0.13 (0.80)	10.92 # (1.32)	100%	0
	Chu et al., 2021 [20]	2.27 (0.88)	NR	12.79 # (1.09)	NR	1 (provisional fracture)
	Cosyn et al., 2011 [21]	NR	−0.34 (0.80)	10.48 # (2.47)	96%	1 (crown loosening)
	Cosyn et al., 2024 [22]	2.02 (1.11)	−0.42 (0.74)	11.37 # (2.19)	100%	NR
	Naji et al., 2021 [26]	3.18 (0.05)	NR	NR	100%	NR
	Puisys et al., 2022 [27]	NR	0.20 (0.38)	12.80 # (1.19)	100%	0
	Seyssens et al., 2020 [9]	1.10 (0.80)	−0.58 (0.60)	10.61 # (1.75)	90%	33% mucositis 5.6% peri-implantitis
**Single-arm data on non-intact alveoli**	Assaf et al., 2017 [16]	2.38 (1.05)	NR	NR	NR	NR
	Borgia et al., 2022 [17]	NR	NR	11.20 # (1.9)	100%	24.2% mucositis
	Liu et al., 2019 [23]	2.31 (1.13)	−0.59 (0.71)	10.58 # (2.47)	100%	5 (wound healing)
	Meijer et al., 2024 [24]	1.18 (0.57)	−0.24 (0.78)	7.40 * (1.40)	100%	20% mucositis 2 (crown loosening and crown fracture)
	Mizuno et al., 2022 [25]	1.70 (0.7)	−0.50 (0.50)	NR	100%	NR
	Pohl et al., 2022 [28]	NR	NR	9.25 # (3.01)	100%	0
	Qian et al., 2023 [29]	2.01 (0.31)	−0.03 (0.17)	9.17 * (0.72)	100%	NR
	Sarnachiaro et al., 2016 [30]	3.00 (NR)	NR	NR	100%	NR
	Sicilia-Felechosa et al., 2020 [31]	NR	NR	12.43 # (2.13)	100%	NR
	Zhao et al., 2023 [32]	2.86 (NR)	NR	11.98 # (0.91)	100%	NR

Negative values indicate vertical midfacial soft tissue level loss (midfacial recession), positive values indicate vertical midfacial soft tissue level gain. * Pink Esthetic Score (PES) based on the modified index generating a score on a total of 10 [33]. # Pink Esthetic Score (PES) based on the original index generating a score on a total of 14 [34]. NR, Not reported.

## Data Availability

The dataset that supports the findings of this study are available from the corresponding author upon reasonable request.

## Supplementary Materials

The following supporting information can be downloaded at: https://www.mdpi.com/article/10.3390/jcm14072462/s1, Supplementary Table S1: Reasons for exclusion. References [42,43,44,45,46,47,48,49,50,51,52,53,54,55,56,57,58,59,60,61,62,63,64,65,66,67,68,69,70,71,72,73,74,75,76,77,78,79,80,81,82,83,84,85,86,87,88,89,90,91,92,93,94,95,96,97,98,99,100,101,102,103] are cited in the supplementary materials.

## Abbreviations

The following abbreviations are used in this manuscript:

IIP	Immediate implant placement
PRISMA	Preferred Reporting Items for Systematic Reviews and Meta-Analyses
PROSPERO	International Prospective Register of Systematic Reviews
NIHR	National Institute for Health Research
CRD	Centre for Reviews and Dissemination
RCT	Randomized Controlled Trial
GBR	Guided Bone Regeneration
MeSH	Medical Subject Headings
PES	Pink esthetic score
RoB 2	Revised Cochrane Risk-of-bias Tool for Randomized Trials
NOS	Newcastle Ottawa Scale
BBT	Buccal bone thickness
DBBM	Deproteinized bovine bone mineral
PRP	Platelet-Rich Plasma
PRF	Platelet-Rich Fibrin
CTG	Connective tissue graft
