# Supplementary material for: A Systematic Review on Immediate Implant Placement in Intact Versus Non-Intact Alveolar Sockets
Source: J Clin Med. 2025 Apr 3;14(7):2462. doi: 10.3390/jcm14072462 (PMC11989472; doi:10.3390/jcm14072462)
Supplement: Supplementary file 1 [file jcm-14-02462-s001.zip › jcm-3540059-supplementary.pdf]

Supplementary Table S1. Reasons for exclusion

Full text not available (a) (in English (b))	Acute infection at site	Too short follow-up (a) or longer follow-up available (b)	Sample size too small	No (consistent) GBR procedure	No differentiation between graft vs no graft	No differentiation between intact vs non-intact	Not reporting on chosen outcome variables	Molar sites included (a) or more IP in the same patient (b)	Not reporting on IIP
Amato and Cracknell (2022) [42] (b)	Elaskary et al. (2021) [43]	Cosyn, De Bruyn, et al. (2013) [44] (b)	Saito et al. (2022) [45]	Cordaro et al. (2009) [46]	Benic et al. (2012) [47]	Chen et al. (2005) [48]	Akimoto and Schuler (2012) [49]	Covani et al. (2008) [50] (b)	Jiang et al. (2018) [51]
Petrungaro (2001) [52] (a)		Hu et al. (2021) [53] (a)	Th Elaskary et al. (2020) [54]	Cosyn, Eghbali, et al. (2013) [55]	Canullo et al. (2009) [56]	Chen et al. (2007) [57]	Arora and Ivanovski (2018) [58]	De Angelis et al. (2011) (a) [59]	Lee et al. (2021) [60]
Rupchandani (2021) (a) [61]		Meijer et al. (2019) (b) [62]	Takai et al. (2017) [63]	Covani et al. (2004) [64]	Kamperos et al. (2016) [65]	Fujita et al. (2021) [66]	Becker et al. (1999) [67]	Juodzbalys and Wang (2007) (b) [68]	Polizzi et al. (2000) [69]
Sun et al. (2020) (b) [70]		Slagter et al. (2016) (b) [71]		Guarnieri et al. (2014) [72]		Grassi et al. (2019) [73]	Bittner et al. (2020) [74]	Noelken, Moergel, Kunkel, et al. (2018) (b) [75]	Veis et al. (2004) [76]
Verdugo et al. (2022) (a) [77]		Slagter et al. (2021) (b) [78]		Lee et al. (2020) [79]		Lee et al. (2012) [80]	Capelli et al. (2013) [81]	Noelken et al. (2016) (b) [82]	
Xie et al. (2019) (b) [83]				Lindeboom et al. (2006) [84]		Naldini et al. (2022) [85]	Cardaropoli et al. (2019) [86]		
Zhang et al. (2020) (b) [87]				Mangano et al. (2013) [88]		Noelken, Geier, et al. (2018) [89]	Cornelini et al. (2004) [90]		
				Östman et al. (2020) [91]		Noelken, Moergel, Pausch, et al. (2018) [92]	Covani et al. (2014) [93]		
				Raes et al. (2011) [94]		Staas et al. (2022) [95]	Kan et al. (2007) [96]		
				Raes et al. (2013) [97]			Koh et al. (2011) [98]		
				Slagter et al. (2017) [99]			Rosa et al. (2014) [100]		
				van Kesteren et al. (2010) [101]			Valentini et al. (2010) [102]		
							Wu et al. (2019) [103]		

References

Akimoto, K. M., and R. F. Schuler. 2012. 'Ridge Width Alteration After Implant Placement Into the Fresh Extraction Socket With Deproteinized Bovine Bone Mineral and Acellular Dermal Matrix', *Clin Adv Periodontics*, 2: 89-95. DOI: 10.1902/cap.2011.110039

Amato, F., and T. J. Cracknell. 2022. 'Single-Tooth Immediate Placement and Provisionalization with Subcrestally Angulated Implants in Sites with Hard and Soft Tissue Facial Dehiscence in the Esthetic Zone: An Observational Study with 2 to 5 Years of Follow-up', *Int J Periodontics Restorative Dent*, 42: e133-e42. DOI: 10.11607/prd.6049

Arora, H., and S. Ivanovski. 2018. 'Evaluation of the influence of implant placement timing on the esthetic outcomes of single tooth implant treatment in the anterior maxilla: A retrospective study', *J Esthet Restor Dent*, 30: 338-45. DOI: 10.1111/jerd.12385

Becker, W., C. Dahlin, U. Lekholm, C. Bergstrom, D. van Steenberghe, K. Higuchi, and B. E. Becker. 1999. 'Five-year evaluation of implants placed at extraction and with dehiscences and fenestration defects augmented with ePTFE membranes: results from a prospective multicenter study', *Clin Implant Dent Relat Res*, 1: 27-32. DOI: 10.1111/j.1708-8208.1999.tb00088.x

Benic, G. I., M. Mokti, C. J. Chen, H. P. Weber, C. H. Hämmerle, and G. O. Gallucci. 2012. 'Dimensions of buccal bone and mucosa at immediately placed implants after 7 years: a clinical and cone beam computed tomography study', *Clin Oral Implants Res*, 23: 560-6. DOI: 10.1111/j.1600-0501.2011.02253.x

Bittner, N., L. Planzos, A. Volchonok, D. Tarnow, and U. Schulze-Späte. 2020. 'Evaluation of Horizontal and Vertical Buccal Ridge Dimensional Changes After Immediate Implant Placement and Immediate Temporization With and Without Bone Augmentation Procedures: Short-Term, 1-Year Results. A Randomized Controlled Clinical Trial', *Int J Periodontics Restorative Dent*, 40: 83-93. DOI: 10.11607/prd.4152

Canullo, L., G. Iurlaro, and G. Iannello. 2009. 'Double-blind randomized controlled trial study on post-extraction immediately restored implants using the switching platform concept: soft tissue response. Preliminary report', *Clin Oral Implants Res*, 20: 414-20. DOI: 10.1111/j.1600-0501.2008.01660.x

Capelli, M., T. Testori, F. Galli, F. Zuffetti, A. Motroni, R. Weinstein, and M. Del Fabbro. 2013. 'Implant-buccal plate distance as diagnostic parameter: a prospective cohort study on implant placement in fresh extraction sockets', *J Periodontol*, 84: 1768-74. DOI: 10.1902/jop.2013.120474

Cardaropoli, D., L. Tamagnone, A. Roffredo, A. De Maria, and L. Gaveglio. 2019. 'Preservation of Peri-implant Hard Tissues Following Immediate Postextraction Implant Placement. Part I: Radiologic Evaluation', *Int J Periodontics Restorative Dent*, 39: 633-41. DOI: 10.11607/prd.4183

Chen, S. T., I. B. Darby, G. G. Adams, and E. C. Reynolds. 2005. 'A prospective clinical study of bone augmentation techniques at immediate implants', *Clin Oral Implants Res*, 16: 176-84. DOI: 10.1111/j.1600-0501.2004.01093.x

Chen, S. T., I. B. Darby, and E. C. Reynolds. 2007. 'A prospective clinical study of non-submerged immediate implants: clinical outcomes and esthetic results', *Clin Oral Implants Res*, 18: 552-62. DOI: 10.1111/j.1600-0501.2007.01388.x

Cordaro, L., F. Torsello, and M. Roccuzzo. 2009. 'Clinical outcome of submerged vs. non-submerged implants placed in fresh extraction sockets', *Clin Oral Implants Res*, 20: 1307-13. DOI: 10.1111/j.1600-0501.2009.01724.x

Cornelini, R., F. Cangini, G. Martuscelli, and J. Wennström. 2004. 'Deproteinized bovine bone and biodegradable barrier membranes to support healing following immediate placement of transmucosal implants: a short-term controlled clinical trial', *Int J Periodontics Restorative Dent*, 24: 555-63.

Cosyn, J., H. De Bruyn, and R. Cleymaet. 2013. 'Soft tissue preservation and pink aesthetics around single immediate implant restorations: a 1-year prospective study', *Clin Implant Dent Relat Res*, 15: 847-57. DOI: 10.1111/j.1708-8208.2012.00448.x

Cosyn, J., A. Eghbali, L. Hanselaer, T. De Rouck, I. Wyn, M. M. Sabzevar, R. Cleymaet, and H. De Bruyn. 2013. 'Four modalities of single implant treatment in the anterior maxilla: a clinical, radiographic, and aesthetic evaluation', *Clin Implant Dent Relat Res*, 15: 517-30. DOI: 10.1111/j.1708-8208.2011.00417.x

Covani, U., A. Barone, R. Cornelini, and R. Crespi. 2004. 'Soft tissue healing around implants placed immediately after tooth extraction without incision: a clinical report', *Int J Oral Maxillofac Implants*, 19: 549-53.

Covani, U., L. Canullo, P. Toti, F. Alfonsi, and A. Barone. 2014. 'Tissue stability of implants placed in fresh extraction sockets: a 5-year prospective single-cohort study', *J Periodontol*, 85: e323-32. DOI: 10.1902/jop.2014.140175

Covani, U., R. Cornelini, and A. Barone. 2008. 'Buccal bone augmentation around immediate implants with and without flap elevation: a modified approach', *Int J Oral Maxillofac Implants*, 23: 841-6.

De Angelis, N., P. Felice, G. Pellegrino, A. Camurati, P. Gambino, and M. Esposito. 2011. 'Guided bone regeneration with and without a bone substitute at single post-extractive implants: 1-year post-loading results from a pragmatic multicentre randomised controlled trial', *Eur J Oral Implantol*, 4: 313-25.

Elaskary, A., M. Meabed, and I. Abd-ElWahab Radi. 2021. 'Vestibular socket therapy with immediate implant placement for managing compromised fresh extraction sockets: A prospective single-arm clinical study', *Int J Oral Implantol (Berl)*, 14: 307-20.

Fujita, Y., T. Nakano, S. Ono, T. Shimomoto, K. Mizuno, H. Yatani, and S. Ishigaki. 2021. 'CBCT analysis of the tissue thickness at immediate implant placement with contour augmentation in the maxillary anterior zone: a 1-year prospective clinical study', *Int J Implant Dent*, 7: 59. DOI: 10.1186/s40729-021-00344-9

Grassi, F. R., R. Grassi, B. Rapone, G. Alemanno, A. Balena, and Z. Kalemaj. 2019. 'Dimensional changes of buccal bone plate in immediate implants inserted through open flap, open flap and bone grafting and flapless techniques: A cone-beam computed tomography randomized controlled clinical trial', *Clin Oral Implants Res*, 30: 1155-64. DOI: 10.1111/clr.13528

Guarnieri, R., R. Placella, L. Testarelli, V. Iorio-Siciliano, and M. Grande. 2014. 'Clinical, radiographic, and esthetic evaluation of immediately loaded laser microtextured implants placed into fresh extraction sockets in the anterior maxilla: a 2-year retrospective multicentric study', *Implant Dent*, 23: 144-54. DOI: 10.1097/id.0000000000000061

Hu, K. S., H. Li, Y. K. Tu, and S. J. Lin. 2021. 'Esthetic results of immediate implant placement in extraction sockets with intact versus deficient walls', *J Dent Sci*, 16: 108-14. DOI: 10.1016/j.jds.2020.06.026

Jiang, X., Y. Zhang, P. Di, and Y. Lin. 2018. 'Hard tissue volume stability of guided bone regeneration during the healing stage in the anterior maxilla: A clinical and radiographic study', *Clin Implant Dent Relat Res*, 20: 68-75. DOI: 10.1111/cid.12570

Juodzbalys, G., and H. L. Wang. 2007. 'Soft and hard tissue assessment of immediate implant placement: a case series', *Clin Oral Implants Res*, 18: 237-43. DOI: 10.1111/j.1600-0501.2006.01312.x

Kamperos, G., I. Zambara, V. Petsinis, and D. Zambaras. 2016. 'The Impact of Buccal Bone Defects and Immediate Placement on the Esthetic Outcome of Maxillary Anterior Single-Tooth Implants', *J Oral Implantol*, 42: 337-41. DOI: 10.1563/aaid-joi-D-16-00004

Kan, J. Y., K. Rungcharassaeng, A. Sclar, and J. L. Lozada. 2007. 'Effects of the facial osseous defect morphology on gingival dynamics after immediate tooth replacement and guided bone regeneration: 1-year results', *J Oral Maxillofac Surg*, 65: 13-9. DOI: 10.1016/j.joms.2007.04.006

Koh, R. U., T. J. Oh, I. Rudek, G. F. Neiva, C. E. Misch, E. D. Rothman, and H. L. Wang. 2011. 'Hard and soft tissue changes after crestal and subcrestal immediate implant placement', *J Periodontol*, 82: 1112-20. DOI: 10.1902/jop.2011.100541

Lee, C. T., E. Sanz-Miralles, L. Zhu, J. Glick, A. Heath, and J. Stoupel. 2020. 'Predicting bone and soft tissue alterations of immediate implant sites in the esthetic zone using clinical parameters', *Clin Implant Dent Relat Res*, 22: 325-32. DOI: 10.1111/cid.12910

Lee, S. R., T. S. Jang, C. S. Seo, I. O. Choi, and W. P. Lee. 2021. 'Hard Tissue Volume Stability Effect beyond the Bony Envelope of a Three-Dimensional Preformed Titanium Mesh with Two Different Collagen Barrier Membranes on Peri-Implant Dehiscence Defects in the Anterior Maxilla: A Randomized Clinical Trial', *Materials (Basel)*, 14. DOI: 10.3390/ma14195618

Lee, Y. M., D. Y. Kim, J. Y. Kim, S. H. Kim, K. T. Koo, T. I. Kim, and Y. J. Seol. 2012. 'Peri-implant soft tissue level secondary to a connective tissue graft in conjunction with immediate implant placement: a 2-year follow-up report of 11 consecutive cases', *Int J Periodontics Restorative Dent*, 32: 213-22.

Lindeboom, J. A., J. W. Frenken, L. Dubois, M. Frank, I. Abbink, and F. H. Kroon. 2006. 'Immediate loading versus immediate provisionalization of maxillary single-tooth replacements: a prospective randomized study with BioComp implants', *J Oral Maxillofac Surg*, 64: 936-42. DOI: 10.1016/j.joms.2006.02.015

Mangano, F. G., C. Mangano, M. Ricci, R. L. Sammons, J. A. Shibli, and A. Piattelli. 2013. 'Esthetic evaluation of single-tooth Morse taper connection implants placed in fresh extraction sockets or healed sites', *J Oral Implantol*, 39: 172-81. DOI: 10.1563/aaid-joi-d-11-00112

Meijer, H. J. A., K. W. Slagter, A. Vissink, and G. M. Raghoebar. 2019. 'Buccal bone thickness at dental implants in the maxillary anterior region with large bony defects at time of immediate implant placement: A 1-year cohort study', *Clin Implant Dent Relat Res*, 21: 73-79. DOI: 10.1111/cid.12701

Naldini, P., D. Torassa, J. L. Calvo-Guirado, and E. F. Bodereau. 2022. 'Evaluation of Contour Augmentation in Immediate Single-Tooth Implants With and Without Flaps in the Anterior Maxilla: A 1-Year Prospective Study', *Int J Periodontics Restorative Dent*, 42: 331-39. DOI: 10.11607/prd.4619

Noelken, R., J. Geier, M. Kunkel, S. Jepsen, and W. Wagner. 2018. 'Influence of soft tissue grafting, orofacial implant position, and angulation on facial hard and soft tissue thickness at immediately inserted and provisionalized implants in the anterior maxilla', *Clin Implant Dent Relat Res*, 20: 674-82. DOI: 10.1111/cid.12643

Noelken, R., M. Moergel, M. Kunkel, and W. Wagner. 2018. 'Immediate and flapless implant insertion and provisionalization using autogenous bone grafts in the esthetic zone: 5-year results', *Clin Oral Implants Res*, 29: 320-27. DOI: 10.1111/clr.13119

Noelken, R., M. Moergel, T. Pausch, M. Kunkel, and W. Wagner. 2018. 'Clinical and esthetic outcome with immediate insertion and provisionalization with or without connective tissue grafting in presence of mucogingival recessions: A retrospective analysis with follow-up between 1 and 8 years', *Clin Implant Dent Relat Res*, 20: 285-93. DOI: 10.1111/cid.12595

Noelken, R., F. Oberhansl, M. Kunkel, and W. Wagner. 2016. 'Immediately provisionalized OsseoSpeed(™) Profile implants inserted into extraction sockets: 3-year results', *Clin Oral Implants Res*, 27: 744-9. DOI: 10.1111/clr.12651

Östman, P. O., S. J. Chu, C. Drago, H. Saito, and M. Nevins. 2020. 'Clinical Outcomes of Maxillary Anterior Postextraction Socket Implants with Immediate Provisional Restorations Using a Novel Macro-Hybrid Implant Design: An 18- to 24-Month Single-Cohort Prospective Study', *Int J Periodontics Restorative Dent*, 40: 355-63. DOI: 10.11607/prd.4467

Petrungaro, P. S. 2001. 'Immediate restoration of dental implants in the aesthetic zone', *Dent Implantol Update*, 12: 89-95.

Polizzi, G., U. Grunder, R. Goené, N. Hatano, P. Henry, W. J. Jackson, K. Kawamura, F. Renouard, R. Rosenberg, G. Triplett, M. Werbitt, and B. Lithner. 2000. 'Immediate and delayed implant placement into extraction sockets: a 5-year report', *Clin Implant Dent Relat Res*, 2: 93-9. DOI: 10.1111/j.1708-8208.2000.tb00111.x

Raes, F., J. Cosyn, E. Crommelinck, P. Coessens, and H. De Bruyn. 2011. 'Immediate and conventional single implant treatment in the anterior maxilla: 1-year results of a case series on hard and soft tissue response and aesthetics', *J Clin Periodontol*, 38: 385-94. DOI: 10.1111/j.1600-051X.2010.01687.x

Raes, F., J. Cosyn, and H. De Bruyn. 2013. 'Clinical, aesthetic, and patient-related outcome of immediately loaded single implants in the anterior maxilla: a prospective study in extraction sockets, healed ridges, and grafted sites', *Clin Implant Dent Relat Res*, 15: 819-35. DOI: 10.1111/j.1708-8208.2011.00438.x

Rosa, J. C., A. C. Rosa, C. E. Francischone, and B. S. Sotto-Maior. 2014. 'Esthetic outcomes and tissue stability of implant placement in compromised sockets following immediate dentoalveolar restoration: results of a prospective case series at 58 months follow-up', *Int J Periodontics Restorative Dent*, 34: 199-208. DOI: 10.11607/prd.1858

Rupchandani, R. 2021. 'Do immediately placed implants have better outcomes when placed with a minimal split-thickness envelope flap?', *Evid Based Dent*, 22: 126-27. DOI: 10.1038/s41432-021-0215-x

Saito, H., S. J. Chu, and D. P. Tarnow. 2022. 'The effect of implant macrogeometry in immediate tooth replacement therapy: A case series', *J Esthet Restor Dent*, 34: 154-66. DOI: 10.1111/jerd.12849

Slagter, K. W., H. J. A. Meijer, D. F. M. Hentenaar, A. Vissink, and G. M. Raghoebar. 2021. 'Immediate single-tooth implant placement with simultaneous bone augmentation versus delayed implant placement after alveolar ridge preservation in bony defect sites in the esthetic region: A 5-year randomized controlled trial', *J Periodontol*, 92: 1738-48. DOI: 10.1002/jper.20-0845

Slagter, K. W., H. J. Meijer, N. A. Bakker, A. Vissink, and G. M. Raghoebar. 2016. 'Immediate Single-Tooth Implant Placement in Bony Defects in the Esthetic Zone: A 1-Year Randomized Controlled Trial', *J Periodontol*, 87: 619-29. DOI: 10.1902/jop.2016.150417

Slagter, K. W., G. M. Raghoebar, N. A. Bakker, A. Vissink, and H. J. Meijer. 2017. 'Buccal bone thickness at dental implants in the aesthetic zone: A 1-year follow-up cone beam computed tomography study', *J Craniomaxillofac Surg*, 45: 13-19. DOI: 10.1016/j.jcms.2016.11.004

Staas, T. A., E. Groenendijk, E. Bronkhorst, L. Verhamme, G. M. Raghoebar, and G. J. Meijer. 2022. 'Does initial buccal crest thickness affect final buccal crest thickness after flapless immediate implant placement and provisionalization: A prospective cone beam computed tomogram cohort study', *Clin Implant Dent Relat Res*, 24: 24-33. DOI: 10.1111/cid.13060

Sun, L., M. M. Yang, J. M. Zhao, X. Zhang, and Z. Qu. 2020. '[Analysis of the hard and soft tissue following immediate and early implant placement in the anterior area of maxilla]', *Zhonghua Kou Qiang Yi Xue Za Zhi*, 55: 857-63. DOI: 10.3760/cma.j.cn112144-20200610-00328

Takai, Y., K. Ouhara, A. Movila, and T. Kawai. 2017. 'Retrospective Case Series Analysis to Evaluate Ridge Augmentation Procedure Applied to Immediate Implant Placement in the Esthetic Zone: Five-Year Longitudinal Evaluation Using Cone Beam Computed Tomography', *Int J Periodontics Restorative Dent*, 37: 521-30. DOI: 10.11607/prd.2958

Th Elaskary, A., Y. Gaweesh Y, M. A. Maebed, S. C. Cho, and M. El Tantawi. 2020. 'A Novel Method for Immediate Implant Placement in Defective Fresh Extraction Sites', *Int J Oral Maxillofac Implants*, 35: 799-807. DOI: 10.11607/jomi.8052

Valentini, P., D. Abensur, J. F. Albertini, and M. Rocchesani. 2010. 'Immediate provisionalization of single extraction-site implants in the esthetic zone: a clinical evaluation', *Int J Periodontics Restorative Dent*, 30: 41-51.

van Kesteren, C. J., J. Schoolfield, J. West, and T. Oates. 2010. 'A prospective randomized clinical study of changes in soft tissue position following immediate and delayed implant placement', *Int J Oral Maxillofac Implants*, 25: 562-70.

Veis, A. A., A. T. Tsirlis, and N. A. Parisis. 2004. 'Effect of autogenous harvest site location on the outcome of ridge augmentation for implant dehiscences', *Int J Periodontics Restorative Dent*, 24: 155-63.

Verdugo, F., T. Laksmana, A. D'Addona, and A. Uribarri. 2022. 'The Cortical Shield for Facial Bone Reconstruction of Severely Damaged Sockets with Simultaneous Implant Placement: A Feasibility Clinical Trial', *Int J Oral Maxillofac Implants*, 37: 1232-43. DOI: 10.11607/jomi.9713

Wu, D., L. Zhou, J. Lin, J. Chen, W. Huang, and Y. Chen. 2019. 'Immediate implant placement in anterior teeth with grafting material of autogenous tooth bone vs xenogenic bone', *Bmc Oral Health*, 19: 266. DOI: 10.1186/s12903-019-0970-7

Xie, Y. T., L. L. Jiang, J. He, C. F. Deng, and B. H. Zhao. 2019. '[Comparison of short-term clinical effect and assessment of influencial factors around single-tooth in the aesthetic area: immediate implant placement versus delayed implant placement]', *Shanghai Kou Qiang Yi Xue*, 28: 148-53.

Zhang, X. X., X. Y. Wu, T. Xia, S. Feng, Q. Hu, Q. Yan, and B. Shi. 2020. '[Evaluation of immediate implant in patients with limited buccal bone wall dehiscence in single upper anterior tooth]', *Zhonghua Kou Qiang Yi Xue Za Zhi*, 55: 831-37. DOI: 10.3760/cma.j.cn112144-20200615-00348
